# Exposure Pathways of Nontuberculous Mycobacteria Through Soil, Streams, and Groundwater, Hawai'i, USA

**DOI:** 10.1029/2020GH000350

**Published:** 2021-04-01

**Authors:** Stephen T. Nelson, Schuyler Robinson, Kevin Rey, Leeza Brown, Norm Jones, Stephanie N. Dawrs, Ravleen Virdi, Grant J. Norton, L. Elaine Epperson, Nabeeh A. Hasan, Edward D. Chan, Michael Strong, Jennifer R. Honda

**Affiliations:** ^1^ Department of Geological Sciences Brigham Young University Provo UT USA; ^2^ Department of Civil and Environmental Engineering Brigham Young University Provo UT USA; ^3^ Center for Genes, Environment, and Health National Jewish Health Denver CO USA; ^4^ Medicine and Academic Affairs National Jewish Health Denver CO USA; ^5^ Division of Pulmonary Sciences and Critical Care Medicine University of Colorado Anschutz Medical Campus Aurora CO USA; ^6^ Department of Medicine Rocky Mountain Regional Denver Veterans Affairs Medical Center Aurora CO USA

**Keywords:** Ground water, groundwater model, losing stream, mycobacterium, surface water

## Abstract

Although uncommon, nontuberculous mycobacterial (NTM) pulmonary infection in the Hawaiian Islands has a relatively high incidence and mortality compared to the mainland U.S. As a result, this study examines the possible geological and hydrological pathways by which NTM patients may become infected, including the environmental conditions that may favor growth and transport. Previously suggested infection routes include the inhalation of NTM attached to micro‐droplets from infected home plumbing systems and aerosolized dust from garden soil. In this study, we evaluate the possible routes NTM may take from riparian environments, into groundwater, into public water supplies and then into homes. Because NTM are notoriously hydrophobic and prone to attach to surfaces, mineralogy, and surface chemistry of suspended sediment in streams, soils, and rock scrapings suggest that NTM may especially attach to Fe‐oxides/hydroxides, and be transported as particles from losing streams to the aquifer on time‐scales of minutes to days. Within the aquifer, flow models indicate that water may be drawn into production wells on time scales (months) that permit NTM to survive and enter domestic water supplies. These processes depend on the presence of interconnected fracture networks with sufficient aperture to preclude complete autofiltration. The common occurrence of NTM in and around streams, in addition to wells, implies that the natural and built environments are capable of introducing a source of NTM into domestic water supplies via groundwater withdrawals. This may produce a persistent source of NTM infection to individuals through the presence of NTM‐laden biofilms in home plumbing.

## Key Points

- Nontuberculous mycobacterial (NTM) are found in soils and biofilms of riparian environments in Hawai'i
- NTM are likely transported from losing streams to aquifers
- Pumped wells draw NTM into culinary water supplies and into homes. NTM are chlorine resistant and outcompete other taxa in home plumbing

## 1 Introduction

The genus *Mycobacterium* includes more than 199 identified species with standing in the nomenclature (Bacterio.net, 2019; Honda et al., 2016; LPSN, 2020), including *Mycobacterium tuberculosis* and *Mycobacterium leprae*, responsible for tuberculosis and leprosy (previously known as Hansen's disease), respectively. Other species are referred to as nontuberculous mycobacteria (NTM), including *Mycobacterium abscessus, Mycobacterium avium*, *Mycobacterium intracellulare,* and *Mycobacterium chimaera* that cause opportunistic pulmonary disease in humans (Falkinham, 2011; Honda et al., 2016).

Human pulmonary infections are thought to occur through environmental exposure to freshwater from municipal distribution systems and soil. Plausible routes of infection are thought to include inhalation of NTM‐laden water droplets dispersed from biofilms that colonize the inner surfaces of showerheads, faucets, ice machines, etc., as well as inhalation of soil aerosols (Centers for Disease Control [CDC], 2019; Falkinham, 2013). In fact, DNA fingerprints of NTM recovered from the lungs of NTM patients and their household showerheads implicate plumbing as a source of exposure (Falkinham, 2011). A recent summary of environmental conditions that harbor NTM can be found in Honda et al. (2018).

In the United States, Hawai'i shows the highest prevalence of NTM lung disease in the nation with the highest age‐adjusted mortality rates (Adjemian et al., 2012; Mirsaeidi, 2014). We have shown that in Hawai'i, clinically relevant NTM are found in home plumbing biofilms such as those that line showerheads, kitchen and bath faucet interiors, as well as soil, and *M. chimaera* is the most frequently identified NTM from Hawai'i environmental and respiratory specimens (Honda et al., 2016). Thus, NTM species found in the lungs of patients are also found in the environment, and the characteristics of water and soil and their movement in Hawai'i may determine the high incidence of NTM.

To begin to understand the possible exposure pathways of NTM in Hawai'i, it is important to understand that the Hawaiian Islands are underlain by fractured basalt lavas which comprise the primary “basal” aquifer systems that supply freshwater. Basalt aquifers are well known to be highly permeable, a characteristic that favors particle transport in the subsurface. The hydraulic gradient in the vicinity of our numerical groundwater flow model is very low, ∼2 × 10^−3^ (Rotzoll & El‐Kadi, 2007), indicative of a large hydraulic conductivity. Depending on rainfall rates, basalt can weather to soil and saprolite comprised of 1:1 clays (kaolinite and/or halloysite), Fe‐oxides/hydroxides (hematite, maghemite, and goethite), and gibbsite. From prior work, NTM have been shown to attach to Fe‐oxides/hydroxides in Hawai'i soil (Glickman, 2020), which may facilitate NTM entry into ground water supplies.

Currently, there is a discernable lack of information available about the factors and environmental interactions that drive the high burden of NTM in Hawai'i. However, the intrinsic hydrophobic nature of NTM and their strong preference to attach to mineral surfaces (e.g., Bolster et al., 2009) make their occurrence relevant to natural and domestic environments, leading to multiple possible exposure pathways. Because NTM counts are higher in distribution systems than immediately downstream of treated water sources, we hypothesize that NTM move from aquifers to surface water to ground water and and into well‐water distribution systems.

**Figure 1:**
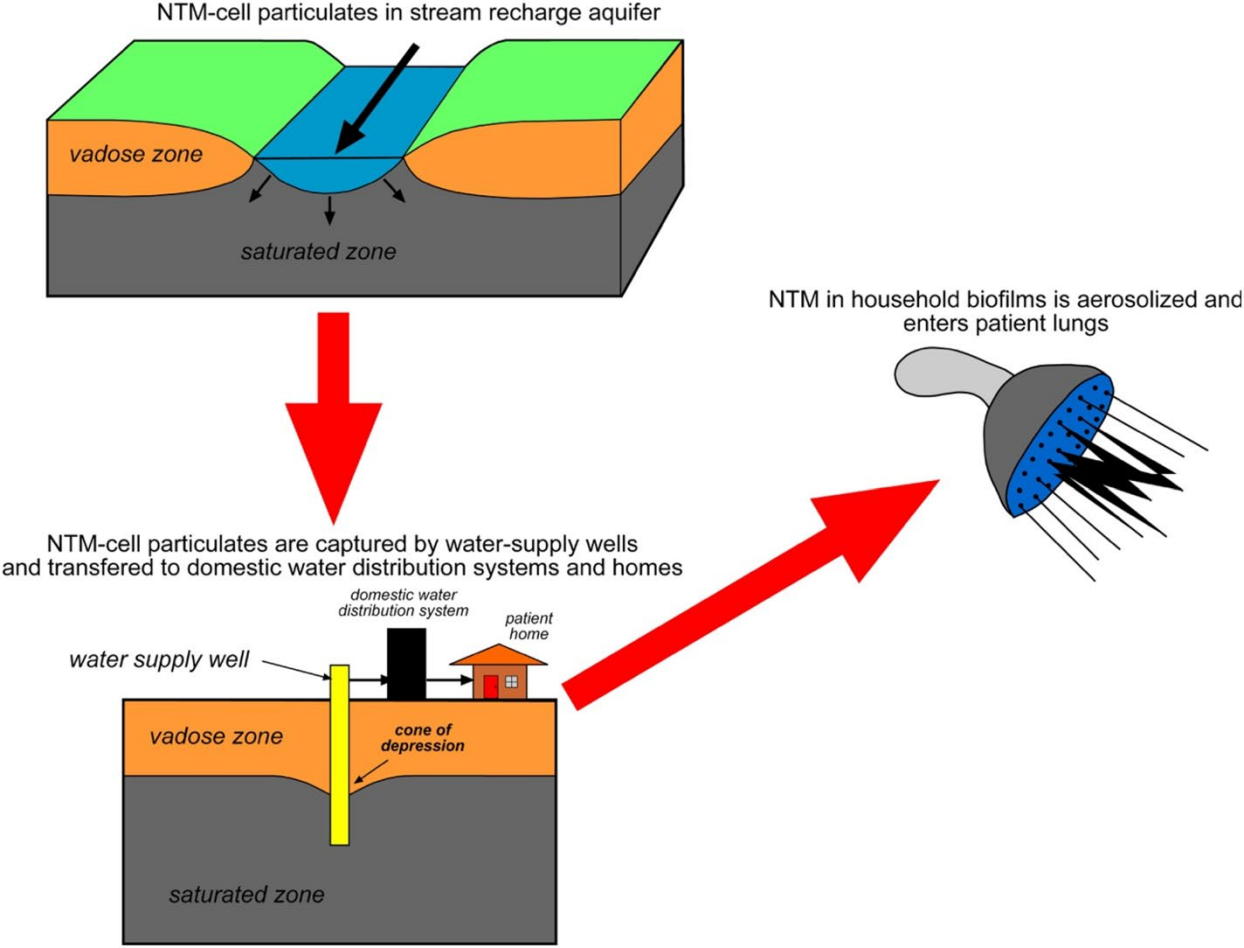
Schematic diagram illustrating pathways for nontuberculous mycobacterial (NTM) movement through the hydrosphere and built environment, possibly leading to patient infection.

This study was performed to evaluate likely or plausible pathways for potential NTM transport through the geosphere and hydrosphere and was motivated by the prevalence of NTM in the soils and biofilms of plumbing in homes across Oahu (Honda et al., 2016). We document NTM in the riparian environments of Oahu and Kauai, analyze minerals in stream sediment to which NTM may bind, and consider losing streams surface and groundwater interactions that may introduce NTM into aquifers, and the potential for NTM transport to water supplies through groundwater withdrawals as summarized in Figure 1.

## 2 Methods

### 2.1 Sample Collection

**Figure 2:**
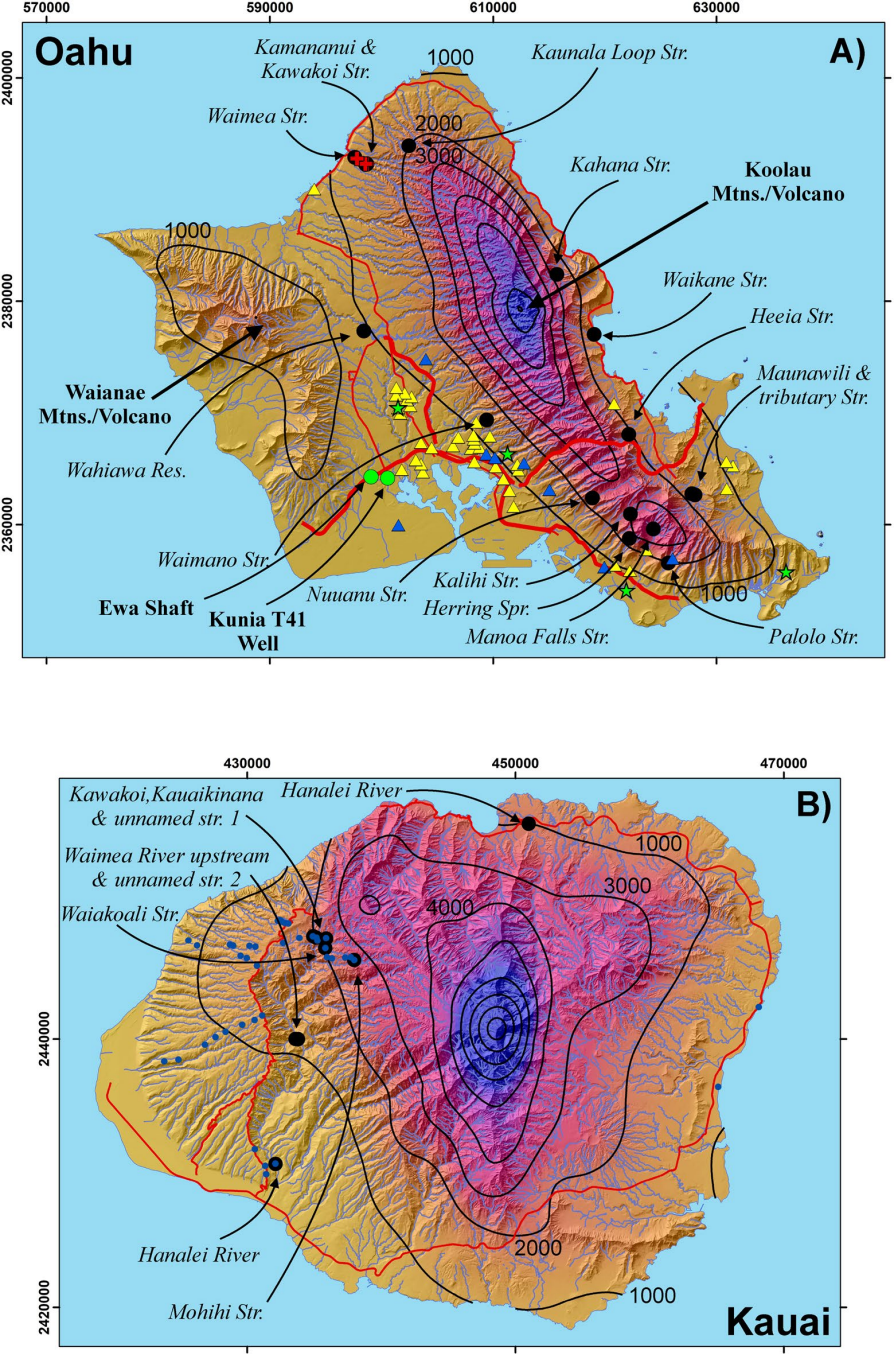
(a) Index map for Oahu indicating stream, spring, and reservoir sampling sites (black circles, labeled). Black circles also indicate the location of streamside soil samples. Also shown are the locations of homes testing positive for nontuberculous mycobacterial (NTM) in DNA swabs (yellow triangles) as well as those testing negative (blue triangles). Locations are approximate to maintain homeowner privacy. Locations for the Ewa Shaft and Kunia T41 Deep Monitoring Well are indicated by green circles. Red crosses indicate the location of gain/loss measurements conducted along a small stretch of the Waimea Stream on August 4, 2018. Green stars indicate the location of dust traps (not discussed here). (b) Index map for Kauai indicating the location of stream samples and soils (blue dots). Red lines are selected major roads. Precipitation contours (1,000 mm annual rainfall interval; Frazier et al., 2016) and draped over a hillshade base map. Coordinates are UTM, NAD83, Zone 4N.

Oahu suspended sediment samples and rock scrapings were collected the week of July 29, 2019. The sampling sites surround the remnants of the Koolau edifice (Figure 2a) and represent a wide variety of climates and accompanying ecosystems from wet to mesic. Kauai stream sediment and rock scrapings were also obtained in July, 2019 during a sampling campaign for dissolved ion chemistry of stream waters. Biofilm samples for microbiological culture were obtained for Oahu in January, February, and August of 2019 and water filtrates were collected in February and August, 2018. Kauai swabs and filtrates were collected in August, 2018 within the Waimea River drainage (Figure 2b). Stream access in Hawai'i is often difficult due to climate (dry areas lack streams), topography, vegetation, and land access restrictions; thus, Kauai sampling was opportunistic.

### 2.2 NTM Processing

For microbial characterization, two activities were performed: (1) swabbing biofilms and (2) obtaining water filtrates. At each sample site, a dual‐tipped Hydraflock Swab (Fisher, 25‐3306‐2H BT) was used to sample biofilms on the surface of boulders or logs just above the water line for NTM microbiological culture at National Jewish Health, Denver. Robinson (2019) provides additional details. Also, ∼0.5–1 L of water was hand filtered through standard 0.2 μm cartridges. In Oahu, acid‐washed bottles were used for filtrate collection. Each bottle was pre‐rinsed with the water to be sampled. The sample was collected and placed in a cooler with ice until filtration could be accomplished in the evening. Unfortunately, the acid‐washed bottles brought to Kauai leaked readily, so we improvised, especially in remote locations, using carbonated‐beverage bottles pre‐rinsed with sample water. These samples are relatively few and cultured bacteria were similar to Oahu samples.

To recover filtrates, snip shears sterilized with 70% ethanol were used to break open the exterior plastic of 0.2 μm syringe filters containing stream water debris. Care was taken not to cut into the filter paper itself. Once the cartridge was open, the membrane was cut into four equal pieces using autoclave‐sterilized razor blades and forceps. The pieces were transferred into a 5 ml screw‐cap tube (Axygen Scientific) containing 2 ml of sterile MilliQ water and vortexed on max for 30 s. Recovery of NTM was performed as we have published (Honda et al., 2016). Briefly, a 450 μl aliquot of neat sample was transferred into a sterile eppendorf tube (Light Labs), to 1% cetylpyridinium chloride was added. The aliquot was vortexed and incubated at room temperature for 30 min. After incubation, 100 μl of disinfected sample was spread onto Middlebrook 7H10 agar plates supplemented with ADNaCl and OADC are incubated at 22°C or 30°C for 21 days. NTM‐like colonies were picked for pure cultures and pelleted for DNA extraction as published by (Epperson & Strong, 2020). NTM were identified by sequencing a region of the RNA polymerase beta subunit (*rpoB*) gene using Sanger sequencing (Quintara Bio). Sequencing results were trimmed for quality control and queried against the National Center for Biotechnology Information (NCBI) GENBank using the BLAST algorithm to compare to *rpoB* strain reference sequences.

### 2.3 Suspended Sediment

#### 2.3.1 X‐Ray Diffraction Analysis

Sediment was retained on 47 mm diameter, 0.45 μm cellulose, and Ag filter membranes. To increase sediment recovery, a subset of samples were centrifuged for 10 min at 4,700 rpm to settle particles <2.5 μm in equivalent spherical diameter at a density of 2.65 g/cm^3^. However, for most samples, filtration was the recovery method of choice given time and logistical constraints in the field, coupled with the remote nature of some sampling sites.

Water was filtered until the Ag membrane clogged (∼500 ml) and then a second membrane was used to double sediment recovery. Ag filters clog more rapidly than cellulose membranes, so additional sediment was recovered on cellulose filters. Sediment from filters was dislodged in an ultrasonic bath for up to ∼1 h in plastic centrifuge tubes. Both cellulose and Ag filters disaggregate somewhat during sonification. Silver filters, however, have the advantage of not contributing an amorphous background to X‐ray diffraction (XRD) patterns. After sediment removal, samples were centrifuged for 4 h at 3,500 rpm to settle particles with an equivalent spherical diameter of 0.6 μm.

Ag filters are useful for the recognition of basal (001) reflections of clays, especially poorly crystalline material, without being masked by the amorphous background of cellulose. However, some samples processed with Ag filters precipitated Ag_2_O, which has a prominent peak at ∼3.2 Å (∼28°), which may interfere with the identification of plagioclase. As cellulose filters retain more sediment, used in combination with Ag membranes, they improve the identification of suspended sediment.

XRD patterns were obtained with a Rigaku Miniflex 600 instrument equipped with a scintillation detector and graphite monochromator using Cu‐radiation from 3 or 5 to 65° 2θ. Patterns were acquired with 0.02° steps with a dwell time of 4–5 s per step. Sediment was suspended in water and loaded onto flat, zero‐background sample holders and allowed to dry at room temperature allowing clays to obtain a preferred orientation with their (001) axes parallel to the sample holder. Although not enough sediment was obtained for quantitative abundances, the diffraction patterns permitted identification of the major suspended phases.

### 2.4 Rock Scrapings

XRD analysis was also performed for Oahu and Kauai rock scrapings obtained from the same locations as suspended sediment. The submerged sides of rocks were scraped with a utility knife/box cutter with disposable blades. Samples were placed in plastic centrifuge tubes, covered with distilled water, and shaken. Material was loaded in suspension with a transfer pipette onto zero background holders. Coarse material in the centrifuge tube was allowed to settle for a few seconds to bias the analysis toward fine grains, similar to suspended sediment. Counting times and ranges were the same as for suspended sediment.

XRD analysis was also conducted on 54 soils taken adjacent to streams on Oahu during sampling in January, February, and July of 2018. Forty samples from Kauai were obtained in July, 2018 and also analyzed. Samples were scanned from 5 to 65° 2θ to facilitate comparison of suspended sediment to soil mineralogy.

### 2.5 SEM Analysis

Material retained on 0.2 μm filters was analyzed from solute‐sampling campaigns conducted in January, 2018. Materials were visualized using an FEI Apreo scanning electron microscope (SEM). X‐ray maps and back scatter or secondary electron images were obtained to characterize sediment mineralogy and grain sizes.

### 2.6 Stream Discharge

Stream flow measurements were made in August, 2018 at three locations along the Waimea and Kamananui Streams located in Waimea Valley, Oahu (Figure 2a). The reader should note that Waimea is a common place name in Hawai'i. Waimea Valley and Waimea Stream on Oahu should not be confused with Waimea Canyon and River on Kauai. Standard stream flow measuring procedures were followed (Rantz, 1982). Velocity measurements were recorded every 0.3 m perpendicular to stream flow.

### 2.7 Groundwater Modeling

**Figure 3:**
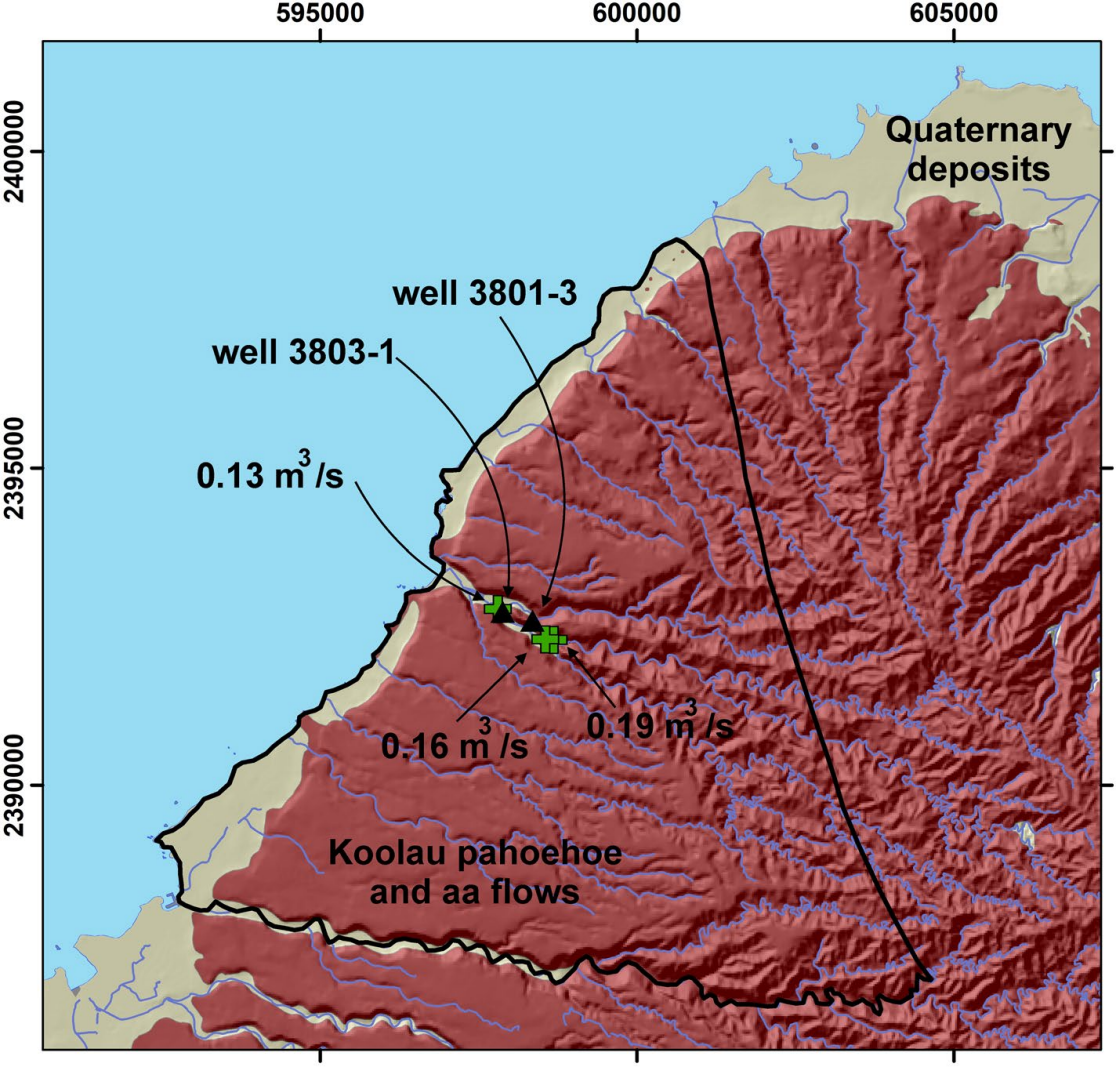
Groundwater model domain including Waimea Stream, Oahu and its tributaries. Water wells are indicated as black triangles, whereas gain/loss measurement sites, including fluxes, are indicated as green crosses. Geology is from Sherrod et al. (2007). Coordinates are UTM, NAD83, Zone 4N.

A groundwater flow model for the Waimea Stream watershed (Figure 3) was modified from an existing Oahu island‐wide model (Rotzoll & El‐Kadi, 2007), which was updated from the Oahu Source Water Assessment Program (Whittier et al., 2010) model by including modern pumping wells, well rates, observed water levels, and land use from 1996 to 2005. Six observation wells and one production well were used in their model. Two other water production wells, 3803‐01 and 3803‐03, are present in the model domain that were not included in the Rotzoll and El‐Kadi (2007) model. Pumping rates of 378 and 757 m^3^/d were used for 3803‐01 and 3803‐03, respectively, in accordance with the maximum water right allotment (UH Manoa, 2012). These wells, however, had a negligible effect on the overall water budget and groundwater surface and were thus not used until after calibration. Additionally, the one production well that Rotzoll and El‐Kadi used to calibrate their model, was not included in our calibration due to negligible effects near the boundary. The model domain is nearly entirely underlain by Koolau pahoehoe and aa flows dipping 3°–10° (Sherrod et al., 2007), and have high hydraulic conductivities of 150–1,500 m/day (Hunt, 1996).

This model was run using the MODFLOW 2000 (Harbaugh et al., 2000; Hill et al., 2000) version in Aquaveo's Groundwater Modeling System software with the Layer Property Flow package. Our use of MODFLOW is consistent with the work of Rotzoll and El‐Kadi (2007) and Whittier et al. (2010). MODFLOW is a continuum model and when applied to bedrock, it relies on the assumption that fracture networks constitute an equivalent porous medium (e.g., Berkowitz and Bear, 1988; Long et al., 1982). We believe this assumption is justified as pahoehoe flows have numerous fractures and aa flows have regularly spaced fractures. Khaleel (1989) noted that as long as the length scale of grids is at least six times the fracture spacing, the assumption of an equivalent porous medium is met. The grid dimensions in the model are much greater than a 1 m upper limit to the spacing of fractures in aa.

Initially, a single layer model was employed for calibration. After calibration, the model was converted to a three‐layer system for particle tracking. The bottom of layers one and two were assigned elevations based on the elevation of well screens of the production wells. The Preconditioned Conjugate Gradient solver was used in all stages of modeling.

Boundary locations were chosen based on aquifer divisions established in previous studies (Mink & Lau, 1990). Specified flux boundaries occur where specified volumes of groundwater are transferred into or out of the model domain. The eastern and southern boundaries of the model were set as specified flux boundaries based on inflows from Rotzoll and El‐Kadi (2007). The coastal boundary was modeled as a specified head condition with an elevation equal to sea level, with accompanying discharges to the ocean (Figure 3).

A major modification to the model domain involved the addition of the Waimea Stream and associated flows. Waimea Stream, as discussed below, loses flow to groundwater. The thickness of the stream bottom sediment was estimated based on well logs and previous studies (Hampton et al., 2004; Hunt, 1996; Lautze & Thomas, 2012; Mink & Lau, 1980; UH Manoa, 2012). Conductance of the river bed sediments to the underlying aquifer was defined as:

(1) $$C=\frac{kA}{M},$$

where *C* is the conductance, *k* is hydraulic conductivity of the river bed sediments, *A* is the plan‐view area of the stream channel available for infiltration (the length of the river multiplied by the channel width), and *M* is the thickness of the river bed sediments. Ephemeral tributaries were represented as drains, where the conductance was adjusted until it approximately matched the volume of water discharging into the aquifer from the stream. The volumetric discharge to groundwater in the perennial stream was determined by the stream flow differences measured in August, 2018.

The goal for modeled heads was to match observation wells within 0.61 m while matching fluxes to ±5% to the values of Rotzoll and El‐Kadi (2007). Six observation wells exist and in the study area and were used for calibration by Rotzoll and El‐Kadi (2007). These same wells (e.g., 3503‐01, 3505‐25, 3604‐01, 3803‐03, 3902‐03, 4101‐03) were also used in the calibration for our model. As mentioned above, after the initial calibration, the model was split into three layers in order to better depict flow paths or capture zones for the two Waimea Valley pumping wells and their specific screen elevations. The addition of multiple layers required the specification of vertical hydraulic conductivity, which is not used in a single layer model. Two trial models were created with differing horizontal to vertical conductivity ratios (i.e., Kh/Kv) of 20 and 200, which are consistent with Souza and Voss (1987) who noted that the horizontal permeability of basalt aquifers may be from one to three orders of magnitude higher than the vertical component. Both models used a porosity value of 5% across the model extent. Additional detail for the model is found in Robinson (2019).

## 3 Results

### 3.1 NTM in Riparian Environments

NTM were recovered from 46% (18/39) of stream water filters from Oahu and Kauai, including the clinically significant species of *M. abscessus, M. porcinum,* and *M. intracellulare*. Two actively purging irrigation water supply wells (Laie Well #1 and #2) were also sampled near Laie, Oahu which recovered *M. abscessus* and *M. triplex*. Ten of 41 (24%) of biofilm swabs also tested positive for NTM species (Table 1). Sample locations for NTM cultures, suspended stream sediment samples, rock scrapings, and stream‐adjacent soils are provided in Table S1.

**Table 1:** *Summary of NTM Taxa Detected on Cultures From 0.2 μm Filters and Biofilm Swabs of Stream Boulders or Logs Just Above Stream Level and Well Water Samples*

Sample name	Locality	Filter NTM taxa	Swab NTM taxa
Oahu samples			
18‐KAR‐01	Manoa Falls Stream	*M. stomatepiae*	**
18‐KAR‐02	Nuuanu Stream	*M. stomatepiae*	*
		*Mycobacterium spp*.	
18‐KAR‐03	Kalihi Stream	*M. stomatepiae*	*
		*M. abscessus*	
18‐KAR‐04	Laie Well #1	*M. abscessus*	*
18‐KAR‐05	Laie Well #2	*	*
18‐KAR‐06	Waimea Stream	**	*
18‐KAR‐07	Kahana Stream	*	*
18‐KAR‐08	Puu Ohia Trailhead***	No filter was collected	**
18‐KAR‐09	Palolo Stream	*M. stomatepiae*	**
18‐KAR‐11	Waimano Stream	*	*
18‐KAR‐12	Wahiawa Reservoir	*	**
18‐KAR‐13	Maunawili Stream	Not analyzed	*
18‐KAR‐14	Maunawili tributary Stream	**	*
18‐KAR‐15	Heeia Stream	*	*
18‐KAR‐16	Waikane Stream	*	*
18‐KAR‐17	Kaunala Loop Trail Stream	**	*
18‐KRM‐01	Manoa Falls Stream	*M. mucogenicum*	*Mycobacterium sp. QIA‐37*
18‐KRM‐02	Nuuanu Stream	*M. stomatepiae*	*
18‐KRM‐03	Kalihi Stream	**	*
18‐KRM‐04	Laie Well #1	*	**
18‐KRM‐05	Laie Well #2	*M. triplex*	**
18‐KRM‐06	Waimea Stream	**	*
18‐KRM‐07	Kahana Stream	*M. intracellulare*	*
		*M. stomatepiae*	
		*M. florentinum*	
18‐KRM‐08	Puu Ohia Trailhead	No filter was collected	*
18‐KRM‐09	Palolo Stream	*M. mucogenicum,*	**
		*Mycobacterium spp*.	
18‐KRM‐11	Waimano Stream	*M. genavense*	*M. chelonae*
18‐KRM‐12	Wahiawa Reservoir	*M. grossiae*	**
18‐KRM‐13	Maunawili Stream	*	*
18‐KRM‐14	Maunawili tributary Stream	**	**
18‐KRM‐15	Heeia Stream	*M. porcinum*	*
18‐KRM‐16	Waikane Stream	*	**
18‐KRM‐17	Kaunala Loop Trail Stream	*	**
18‐KRM‐18	Herring Springs	*M. florentinum*	*Mycobacterium sp. QIA‐37*
			*Mycobacterium sp. FI‐09183*
18‐KRM‐19	Kamananui Stream	*M. stomatepiae*	**
		*M. abscessus*	
18‐KRM‐20	Kaiwikoele Stream	*M. florentinum*	*
		*M. lentiflavum*	
		*M. shigaense*	
		*M. stomatepiae*	
		*M. triplex*	
Kauai samples			
18‐KAU‐19	Waiakoali Stream	*	*M. franklinii*
18‐KAU‐20	Waiakoali Stream	*	*M. porcinum*
18‐KAU‐25	Kawaikoi Stream	**	*M. porcinum*
18‐KAU‐26	Mohihi Stream	*M. parmense*	No swab was collected
		*Mycobacterium sp. EPM10906,*	
		*M. porcinum*	
18‐KAU‐27	Unnamed stream 1	*	*Mycobacterium sp. Strain 38 B,*
			*Mycobacterium sp. EPM10910*
18‐KAU‐34	Kauaikinana Stream	*M. triplex*	*M. porcinum*

*Note*: Taxa were identified by DNA sequencing.

*Nothing grew when cultured, or cultured material was not bacterial.

**Cultured material failed to produced identifiable taxa.

***Swab biofilm samples only. Site is not adjacent to a stream.

### 3.2 Suspended Stream Sediment Analyses

**Figure 4:**
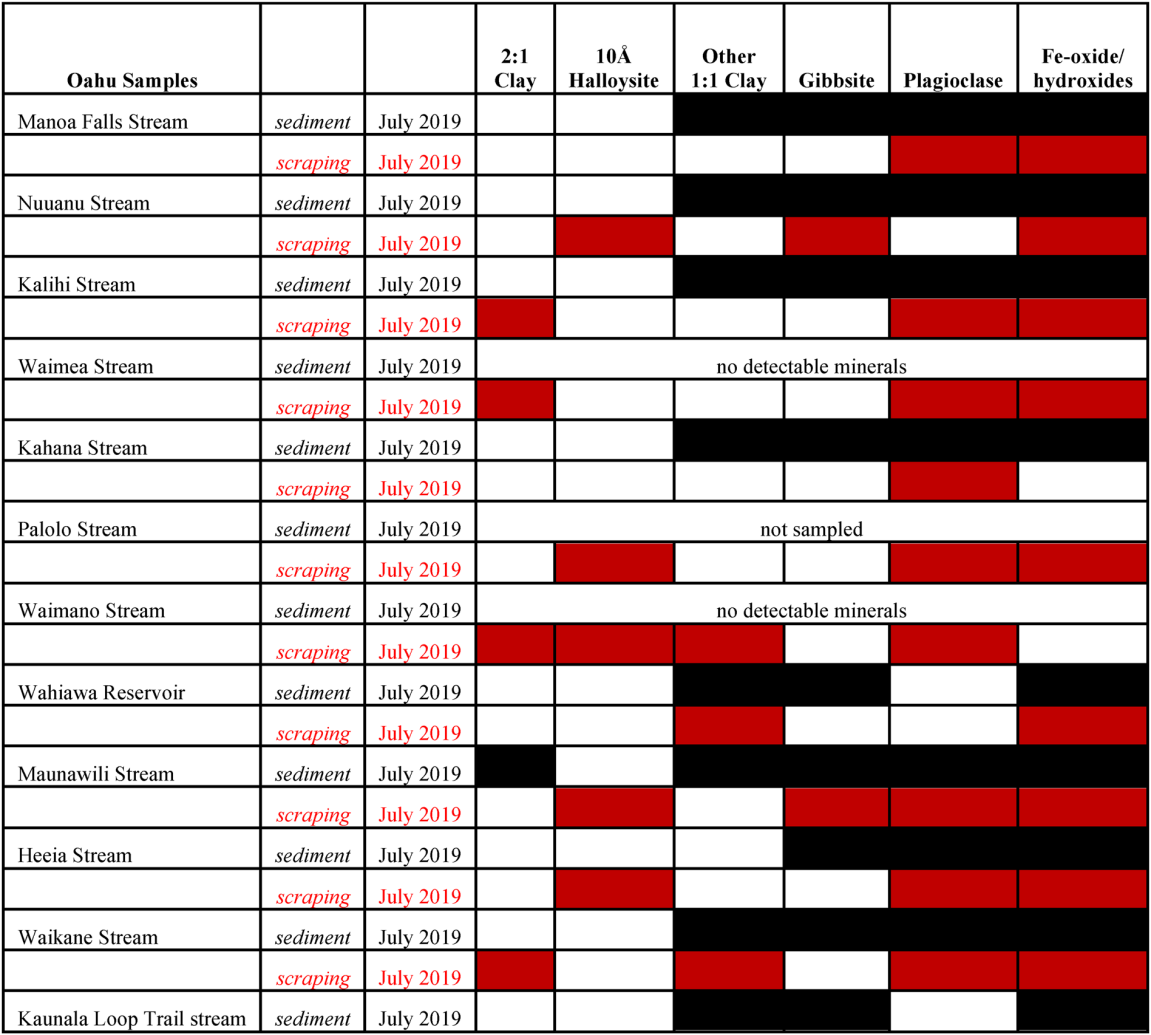
Summary matrix of the mineralogy of selected stream sediment and rock scrapings for Oahu. Filled cells indicate that the mineral was present.

**Figure 5:**
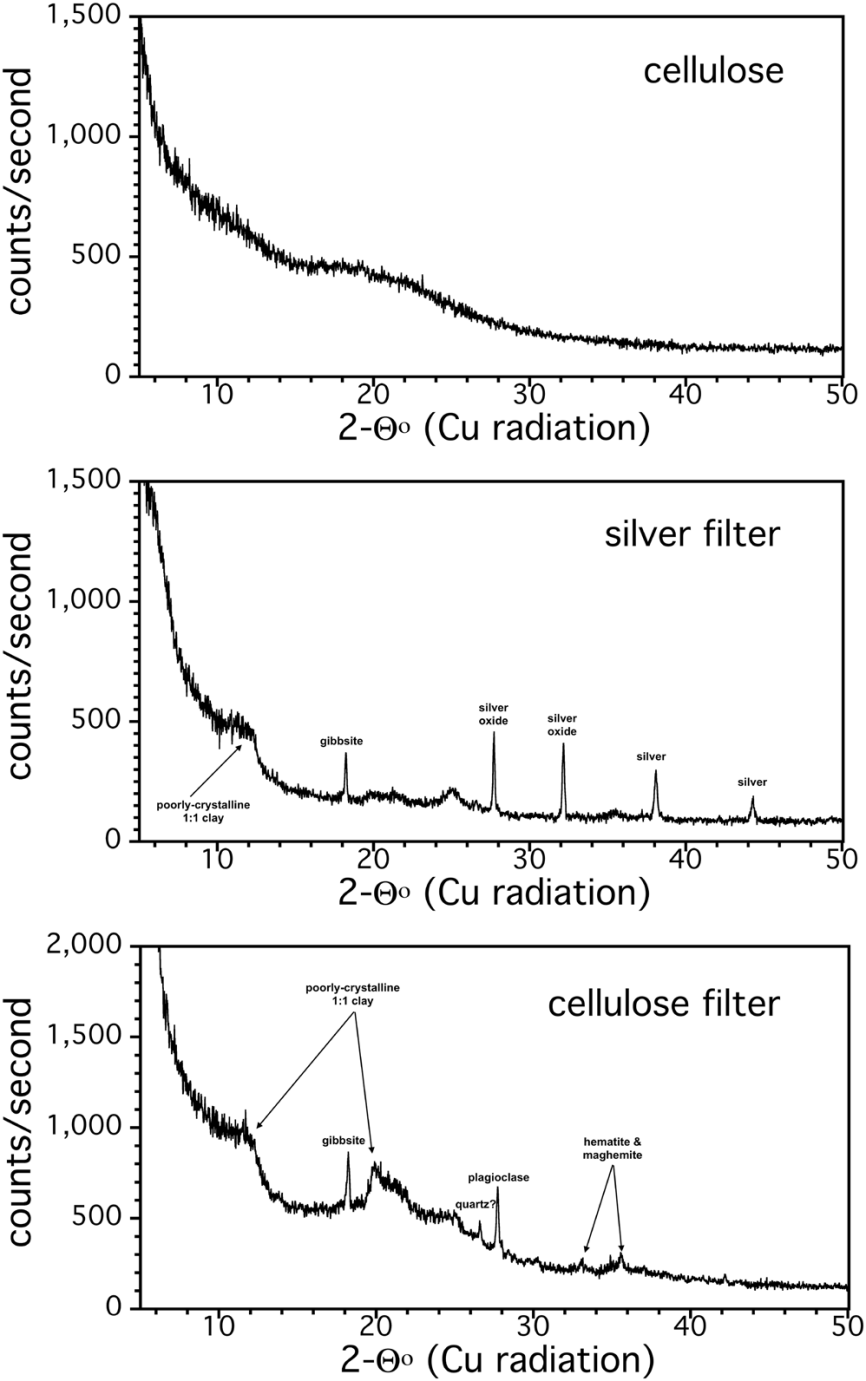
Representative X‐ray diffraction (XRD) pattern for cellulose disaggregated from a filter during sonification (top). Sediment from Maunawili Stream, Oahu retained on silver and cellulose filters are also shown (middle and bottom).

Quartz was present in a few stream samples. The (−112) reflections of plagioclase and gibbsite may interfere with the diagnostic (011) and (101) peaks of quartz. However, in some instances its presence is unambiguous due to the large relative size of the quartz peaks. Gibbsite was an important phase within suspended stream sediment (Figure 4), its 18.16° (002) reflection being diagnostic. However, the small (111) reflection of maghemite, a common phase in Fe‐rich soils of Hawai'i (e.g., Nelson et al., 2013), is close to the gibbsite (002) reflection. Gibbsite was assumed to be absent in cases where the maghemite (111) reflection appeared to have the requisite relative intensity compared to its other peaks. Sample diffraction patterns of a typical Oahu stream filtrate are shown in Figure 5, including the amorphous background contributed by cellulose filters as well as the contribution of AgO_2_ peaks from silver filter membranes.

**Figure 6:**
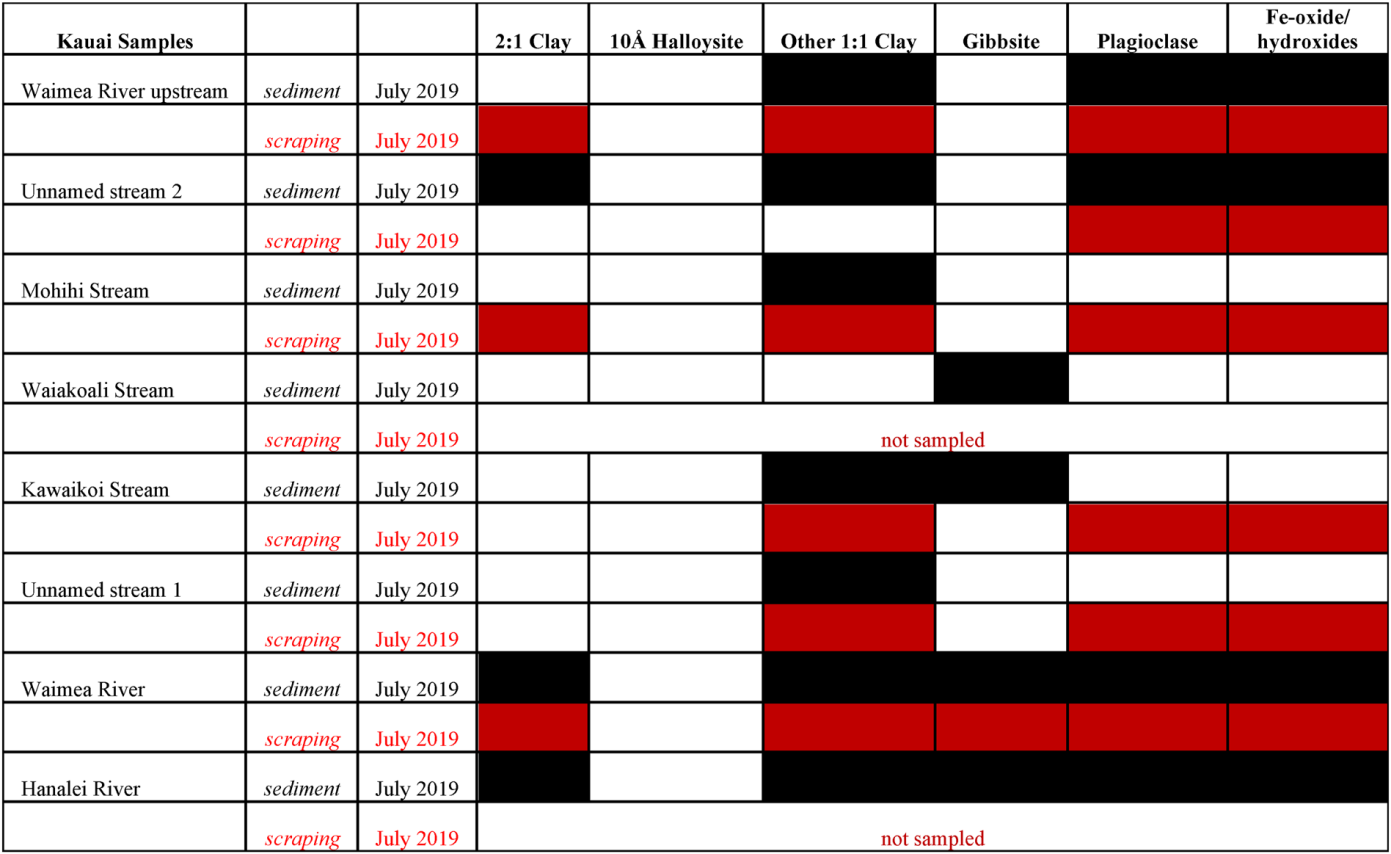
Summary matrix of the mineralogy of selected stream sediment and rock scrapings for Kauai. Filled cells indicate that the mineral was present.

All streams sampled (12/12) contained suspended Fe‐oxide/hydroxides, typically hematite and maghemite. Clay minerals include “other 1:1 clay” (7 Å; kaolinite ± halloysite) (Figure 4), that occurs in most Hawaiian streams. The use of “other” 1:1 clay sets suspended clays apart from rock scrapings, where 10 Å halloysite is present in half of the Oahu samples (Figures 4 and 5), as well as 2:1 clays, which are also present in some rock scrapings but rarely in stream sediment. Gibbsite was common (∼90%) along with plagioclase (∼60%). Samples from Kauai contained a similar suspended sediment mineralogy to that of Oahu, although gibbsite was less common (Figure 6).

### 3.3 X‐Ray Diffraction of Rock Scrapings

**Figure 7:**
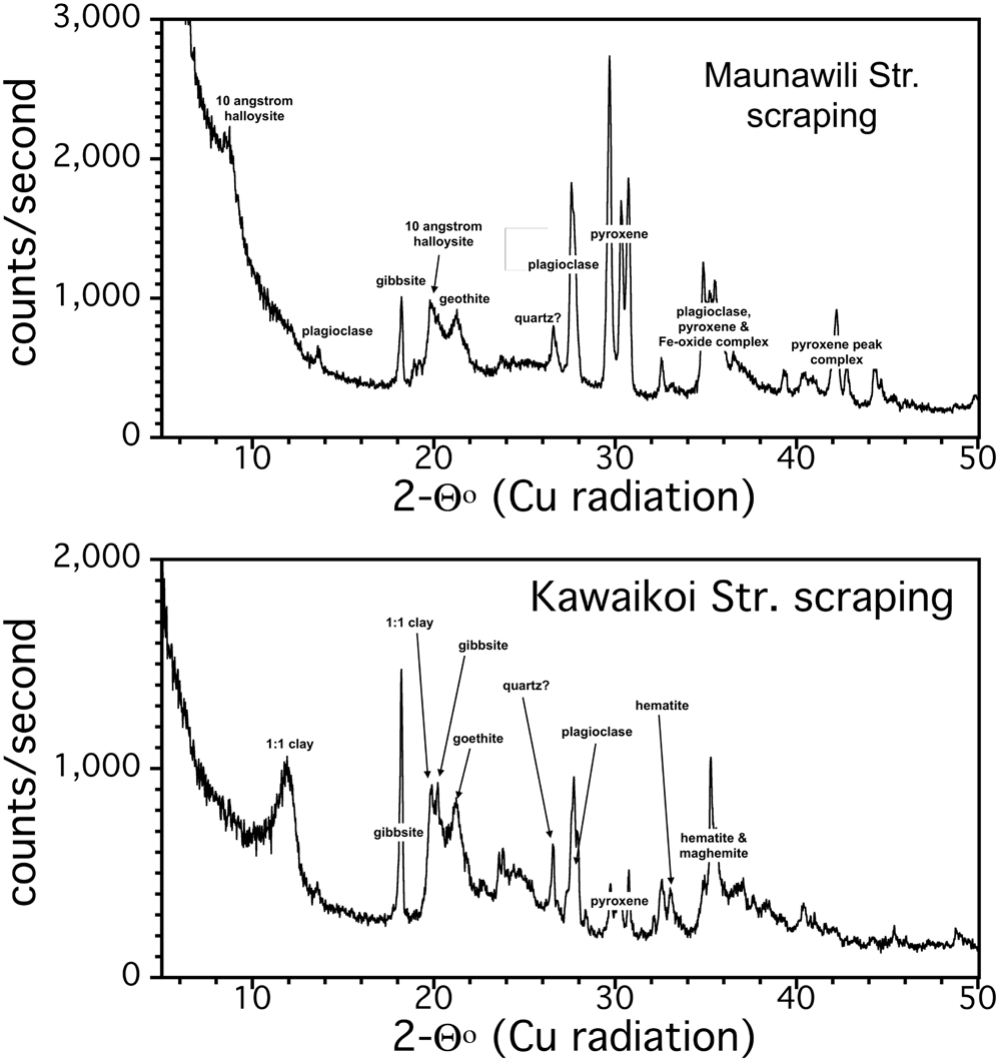
Representative X‐ray diffraction (XRD) patterns for boulder scrapings from Maunawili Stream, Oahu and from Kawaikoi Stream, Kauai.

Rock scrapings sampled alteration rinds, which are relatively thin due to the remobilization and mechanical abrasion of the boulders during episodes of high stream flows. Figure 7 presents representative XRD patterns of boulder scrapings. Beneath the alteration rind, primary plagioclase and pyroxene were also detected. Magnetite was likely present, but its peaks are masked by hematite and especially maghemite and are difficult to resolve.

On Oahu, 13 rock scrapings contained a mixture of primary and alteration minerals. Plagioclase and Fe‐oxides/hydroxides were present in ∼85% of samples, with 1:1 clays and 2:1 clays (∼30%) as well as gibbsite (∼25%). Most surprising, however, was ∼50% of scrapings contained 10 Å halloysite (Figure 4), which does not appear in stream sediment.

Six rock scrapings from Kauai contained a mixture of primary igneous and alteration minerals. All samples contained plagioclase and Fe‐oxides/hydroxides. 1:1 clays were present in five samples, 2:1 clays were present in three samples and gibbsite in one (Figure 6). No 10 Å halloysite was observed.

### 3.4 X‐Ray Diffraction of Soils

Soil sampled adjacent to streams on Oahu showed a typical mineral assemblage of 1:1 clay and Fe‐oxides/hydroxides. 1:1 clays, however, were not universally present, occurring in ∼85%–90% of samples. Presumably, the Al‐reservoir in the remaining ∼20% is an amorphous phase like allophane or imogolite. Only about 20% of soils clearly contained gibbsite, a much smaller fraction than in suspended sediment. Remnant plagioclase was present in ∼50%–60% of soils. 10 Å halloysite occurred in just one sample. The soil of Kauai also contained an assemblage of 1:1 clay and Fe‐oxides/hydroxides, with ∼8% bearing 2:1 clay. Like Oahu, 1:1 clays were not universally present, occurring in ∼80% of samples. Plagioclase was present in only one of 40 samples. The age of the volcanic substrate on Kauai is twice that of Oahu, permitting the more complete consumption of primary igneous minerals like plagioclase in nearly all soils (Sherrod et al., 2007).

### 3.5 Elemental Mapping of Suspended Sediment

**Figure 8:**
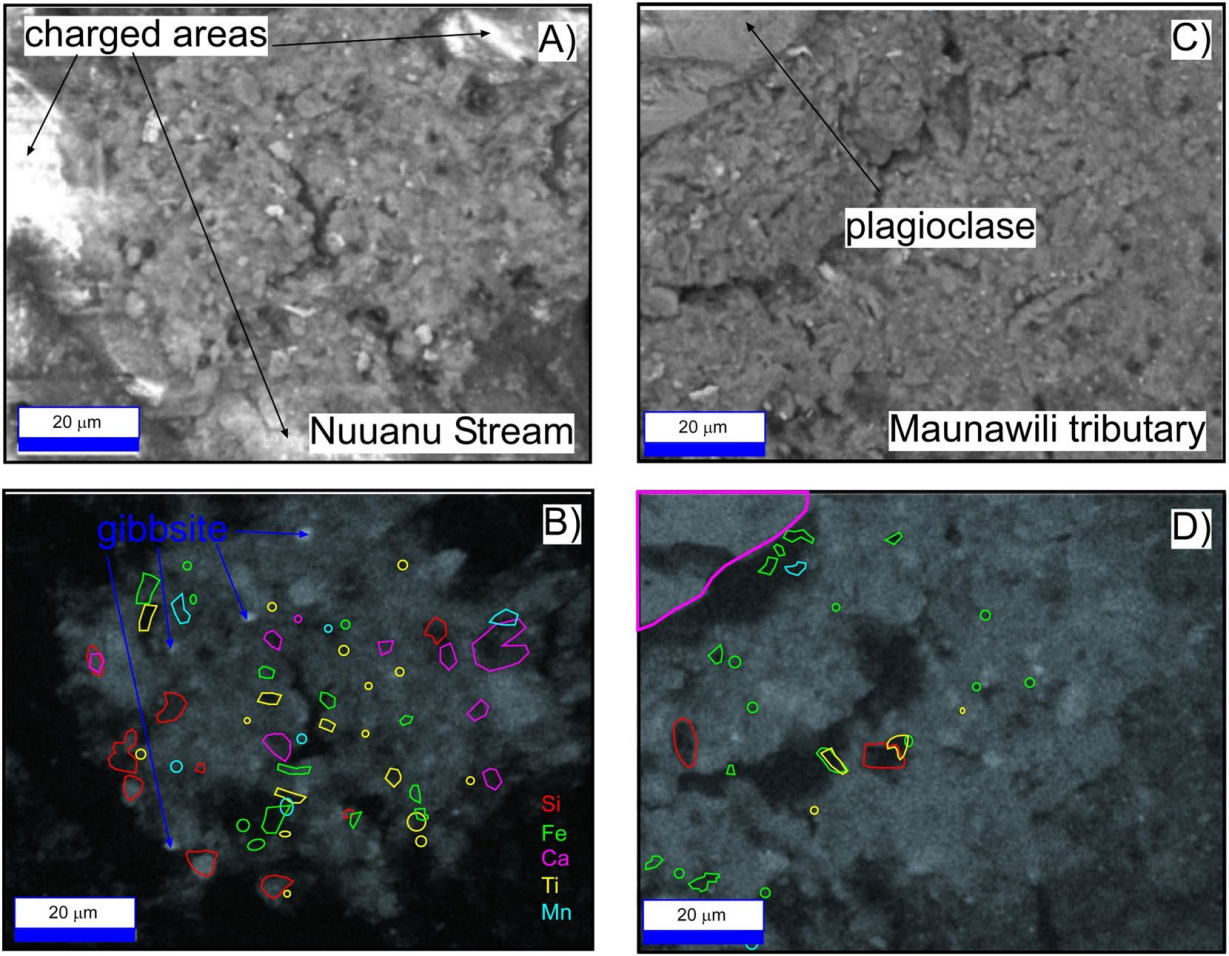
Back‐scatter electron (A and B) for samples from the Kalihi Stream, Oahu, and Maunawili tributary stream, respectively. (C and D) Are Al X‐ray maps corresponding to the samples above them. Polygons represent areas of high concentration for the elements indicated as determined from X‐ray maps not shown. See text for discussion.

To confirm the identity of phases in XRD patterns, energy‐dispersive X‐ray maps were created for flocculated suspended sediment retained on filters for two representative samples (Figure 8). Back‐scatter electron images reflect the average atomic number (*Z*) and grain size. Ignoring light‐colored areas resulting from charge accumulation (Figure 8a), high average *Z* (i.e., bright, Fe‐oxide/hydroxide) minerals are small, dispersed, and represent a small but pervasive fraction of the mass. Overall, the sediment on the filters in Figures 8a and 8c have minerals that are very fine grained, typically <5 μm.

Figures 8b and 8d show X‐ray maps of Al abundance in the Nuuanu Stream and a tributary to the Maunawili Stream, respectively (Table S2). Superimposed are polygons representing bright spots (high elemental abundance) from X‐ray maps (not shown), including Si, Fe, Ca, Ti, and Mn. The Si X‐ray maps resemble Al maps as this element is dispersed throughout the mineral agglomerates in clay. Some Si‐polygons, however, occur in Al‐free areas and clearly represent quartz. This is especially true in Figure 8d where two quartz grains (red polygons) occur where Al is absent. Other Si‐polygons with faint Al backgrounds may also represent quartz, but with fluorescent Al X‐rays being excited by the electron beam from around and beneath small quartz grains.

Ca‐polygons (magenta) are located within areas with subdued Al‐fluorescence, likely representing plagioclase, which contains stoichiometric Al, but at a proportion much less than that of 1:1 clay. This is especially clear in Figures 8c and 8d where a plagioclase grain, complete with cleavage, occupies the upper‐left corner of the image. Some Fe‐polygons overlap with Ti‐, indicative of a solid solution Fe‐Ti oxide like ilmenite. Other Fe‐ and Ti‐, and Mn‐polygons, represent discrete phases.

### 3.6 Stream Flows

On August 4, 2018, flow measurements were taken at three sites in the Waimea Stream (Oahu; Figure 3) at base flow conditions. Over the course of ∼1 km, stream flows decreased from 0.19 to 0.13 m^3^/s, indicating significant losses to the underlying aquifer. Elevations at these sites range from ∼10 to ∼30 m, such that the top of the underlying freshwater lens is not far below the stream.

### 3.7 Groundwater Modeling

The goal for calibrating the groundwater flow model for the Waimea drainage, Oahu (Figure 3) was to replicate the flow budget generated by Rotzoll and El‐Kadi (2007) while calibrating heads to observed water levels used in their model along with measured stream losses. Robinson (2019) indicated that the percent differences between their model and ours is less than 2%. Models were exercised for *K*_h_/*K*_v_ (horizontal: vertical hydraulic conductivity) values of 20 and 200 based on published ranges (Rotzoll & El‐Kadi, 2007; Souza & Voss, 1987). The model with a *K*_h_/*K*_v_ value of 20 produced somewhat better agreement between calculated and observed groundwater elevations.

**Figure 9:**
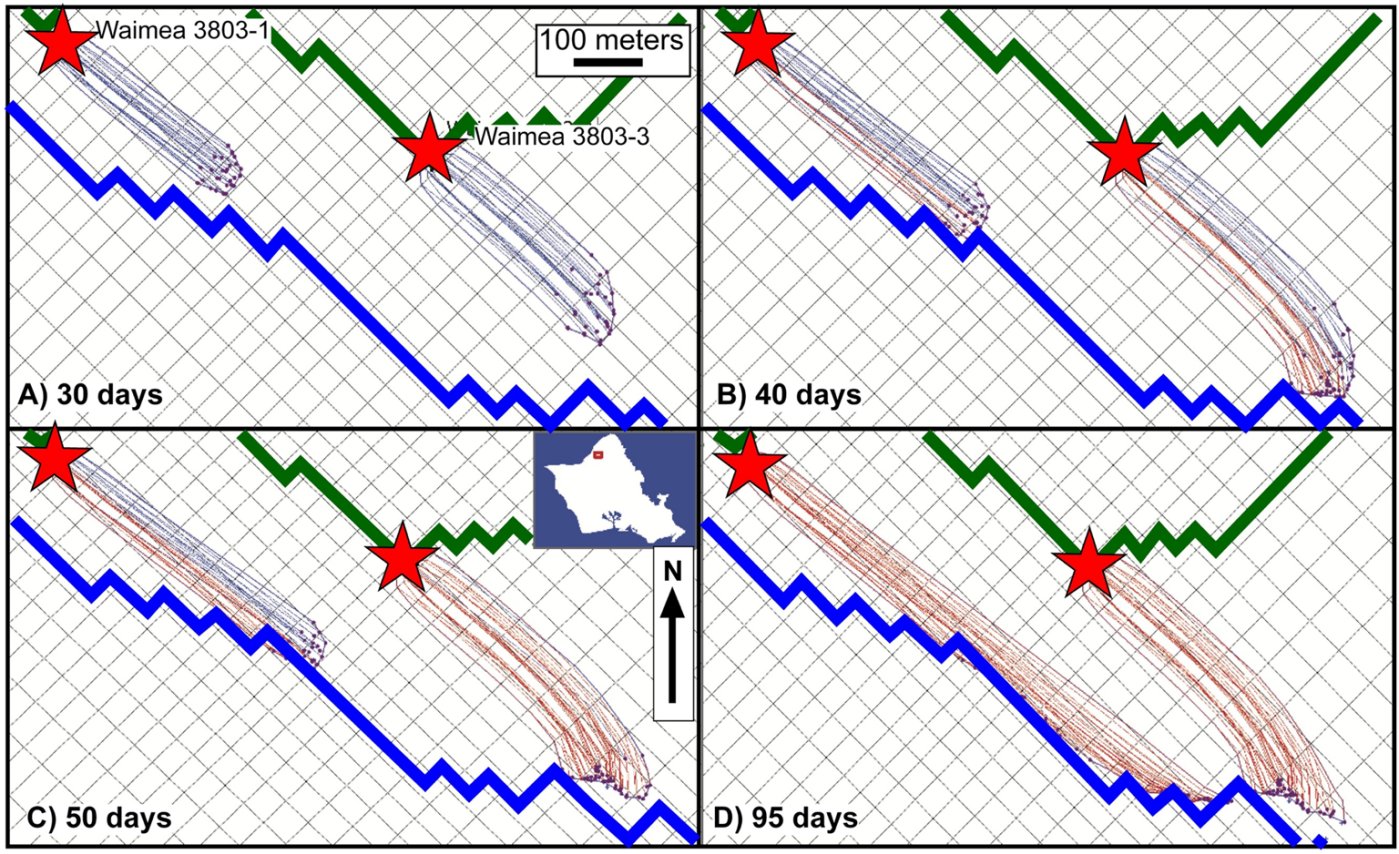
Particle tracking calculations, superimposed on the model grid, from the results of groundwater modeling (Robinson, 2019). Blue and green bold lines, represent perennial (Waimea Stream) and intermittent streams, respectively. Well locations (red stars) are indicated. Fine blue lines indicate paths that do not draw water from a perennial stream in the time indicated, whereas fine red lines indicate paths originating at streams.

Particle tracking was implemented to visualize the potential for, and time scales of, connections between the streams and the production wells in the model domain. Results of particle tracking calculations are presented in Figure 9 for four time intervals. The *K*_h_/*K*_v_ = 20 model showed that after 40 days, the first particles reached well 3803‐01. After 95 days, nearly all water pumped from both wells could be traced back to the streams, with flow paths of 440 and 700 m for wells 3803‐03 and 3803‐01, respectively, where stream water is initially lost to the aquifer and then transported laterally down‐gradient to the wells. A higher value of *K*_h_/*K*_v_ would permit even faster transport to wells. Robinson (2019) provides additional detail for groundwater modeling.

## 4 Discussion

In this study, we evaluate the evidence for the potential of NTM transport from riparian environments into aquifers and into groundwater distribution systems, a process we have outlined in conceptual form (Figure 1). All of our stream filtrates for NTM and stream sediment mineralogy were obtained at or near base flow. By contrast, overland flow to streams would greatly increase sediment abundances as well as NTM numbers in streams if NTM were resident in adjacent soils. In this sense, we present a case for the movement of NTM into and through aquifers where the number of NTM cells introduced are comparatively low.

### 4.1 Assumptions

As noted in the Section 1, our purpose was to evaluate the movement of NTM along the pathways summarized in Figure 1. Although the presence of NTM can be demonstrated in riparian zones as well as homes (Honda et al., 2016), it is only possible to make an inductive case that the movement of NTM through the vadose and saturated zones is likely. To do this, we have to make two important assumptions. First, that NTM cells or cell‐mineral pairs are not completely filtered in fracture networks. Second, that fractured basalts closely approximate an equivalent porous medium in MODFLOW calculations, an assumption that is met according to the criterion of Khaleel (1989). Much of the discussion that follows also addresses these assumptions.

### 4.2 NTM in Riparian Environments

There has been little prior characterization of the biota of Hawai'i groundwater, although one recent study has documented diverse bacterial communities on Oahu to the phylum or class level (Kirs et al., 2020). They noted that *Actinobacteria*, the phylum to which NTM belong, are present in nearly all groundwater, albeit at a relatively low mean abundance (2.2%) but are a major constituent (26%) of the soil biome. Cell counts in other fractured basalt aquifers range from 10^4^ to >10^7^ cells/ml (Colwell & Lehman, 1997; Guðmundsdóttir et al., 2019; Lehman et al., 2004; Zheng & Kellogg, 1994) and seem to be unaffected by hydraulic conductivity (Colwell & Lehman, 1997), with a very strong tendency for bacteria to attach (∼99% of biomass; Lehman et al., 2001). Thus, it is expected that a significant number of bacteria are available to enter the well bores and water supply systems during pumping.

As documented in the Results, NTM, including clinically significant species, were widespread in riparian environments as well as springs, wells, and the Wahiawa Reservoir of Oahu (Table 1). The recent study by Robinson (2019) also reported that five of seven (71%) of swabs collected from the surface of boulders or logs on Oahu were NTM positive, and included the isolation of *M. chimaera*, *M. avium*, and *M. chelonae*. NTM are clearly established in these environments and as such are available for transport along the conceptual pathways outlined in Figure 1.

### 4.3 Attachment Properties of Minerals in Streams

Because NTM prefer to attach to surfaces, we evaluated the surface properties of minerals that are likely to enhance or retard the attachment of bacteria. Considerable research has shown that bacteria tend to bind to the surface of minerals in the order of Fe‐oxides/hydroxides > Al‐oxides/hydroxides (gibbsite) > clay > quartz (Hong et al., 2018; and references therein), and all of these mineral groups are abundant in Hawai'i streams except quartz, which appeared in a few samples. The presence of quartz in Hawai'i may seem surprising to the earth scientist, so we have documented its presence, but it is not considered further with respect to NTM transport given its low tendency to bind to NTM. Nelson et al. (2013) and Porder et al. (2007) discussed the origin of quartz in Hawai'i soil and saprolite.

A number of mechanisms have been proposed for the attachment of microbes to mineral surfaces, including the role of extracellular proteins (e.g., Marshman & Marshall, 1981) or other organic coatings (Taylor & Gulnick, 1996), as well as chemotaxis, double‐layer effects, and cell‐surface hydrophobicity (Marshall, 1975). In addition to hydrophobicity in NTM, electrostatic charge differences are favorable in Hawai'i. The point zero charge (PZC) of a mineral is equivalent to the pH at which there is no surface charge. Where the PZC is greater than solution pH, the mineral will have a net positive charge, which may enable the negatively charged surface of NTM to bind.

The PZCs for gibbsite, goethite, and hematite are 9.27 ± 1.63 (*n* = 8), 7.94 ± 0.87 (*n* = 21) and 8.01 ± 1.07 (*n* = 12), respectively (Kosmulski, 2004, 2006, 2009, 2011; Tan et al., 2008; Tschapek et al., 1974). These values are often higher than measured stream pH in this study (7.07 ± 0.70; *n* = 41 for Oahu; 7.22 ± 0.71; *n* = 6 for Kauai), suggesting that suspended minerals in Hawai'i streams tend to be positively charged. A simple simulation where 1,000 random numbers with a gaussian distribution were generated around the reported means for stream pH and 1σ uncertainties (Oahu only) and the PZCs for gibbsite, goethite, and hematite suggest that these these minerals will have positive surface charges 88.6%, 77.5%, and 76.5% of the time, respectively. Abundant minerals with favorable surface charge are present for the attachment and transport of mineral‐NTM particulate pairs, and the recent results of Glickman et al. (2020) indicate that hematite‐NTM colloids may be particularly important.

### 4.4 Physical Transport of NTM

#### 4.4.1 Transport in the Vadose Zone

Considering the presence of suspended sediment that may bind NTM, we considered whether it is physically reasonable that mineral‐bacterial pairs may be transported: (a) from losing streams to the aquifer, and (b) from the aquifer to wells and water supply systems. However, the inoculation of water supplies seems likely given the presence of significant numbers of bacteria and diverse taxa represented.

Scholl et al. (1995) noted that the high permeability of Hawai'i basalt often results in the absence of perennial streams, even in areas of high rainfall. Near Hilo, Hawai'i, perennial streams are generally absent despite a mean rainfall of ∼2 m/yr. Clearly, water rapidly infiltrates to recharge basalt aquifers, including from streams with losing stretches. Attached or unattached NTM could then be transported to the aquifer provided a fracture pathway exists with sufficient aperture to prevent size exclusion or filtration of bacteria.

Rosa (2017) provided strong empirical evidence for a relationship between storms, surface water flows, and groundwater levels in the basalt aquifer of southwestern Oahu. Such a relationship is self‐evident, but that study showed a rapid response of groundwater elevations to storms and accompanying surface water flows, where the rapid response suggests the presence of open fracture networks.

**Figure 10:**
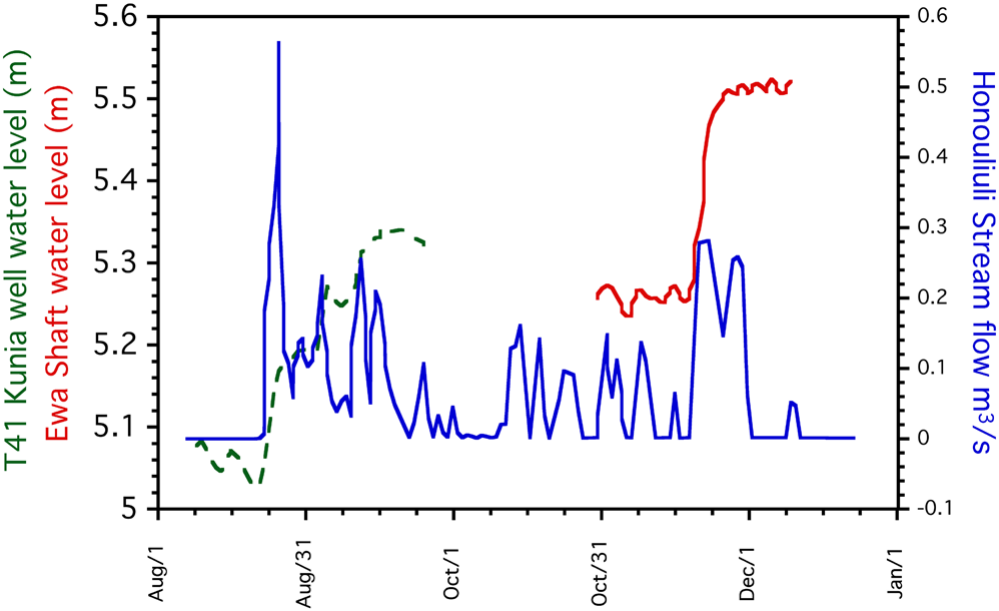
Correlation of intermittent stream flows in the Honouliuli Stream to water levels in the T41 Kunia well and the Ewa Shaft during the latter half of 2016. The well and shaft locations are documented in Figure 1A. Modified from Rosa (2017).

In Hawai'i, most drinking water is supplied by wells or shafts tunneled into the top of the aquifer (Gingerich & Oki, 2000). The Ewa Shaft and the nearby Kunia T‐41 deep monitoring well (Figure 2a) exhibited very rapid (∼24 h) rises of about 0.20–0.25 m in water levels following a series of storms in September and November 2016 that were accompanied by surface flows in the intermittent Honouliuli Stream (Figure 10). The increase in water levels was rapid after the first storm event in each period. The ground surface elevations of the Ewa Shaft and Kunia T‐41 well are 49 and 26 m, respectively. Thus, the water must rapidly pass through a vadose zone of similar thickness. In fact, the motivation for Rosa's (2017) study was concern by the Honolulu Board of Water Supply for the transport of bacteria to the aquifer by losing streams.

A recent study in American Samoa (Shuler et al., 2019) noted that following major rainfall events, turbidity and bacterial numbers (*E. coli* and total coliforms) increased in wells completed in fractured basalt, with breakthrough times of 37 ± 21 h and 18–63 h for particulate matter and cells, respectively. Estimated travel distances through a 20–30 m thick vadose zone, similar to coastal areas of Oahu, were 225–1,000 m, similar to the transport lengths in the groundwater flow model. The analog studies of Rosa (2017) and Shuler et al. (2019) demonstrate that connections between losing streams and the vadose zone to aquifers and water supply wells through fractured basalt can be rapid.

As noted in this study, flows in the Waimea Stream, Oahu (Figures 2a and 3) underwent ∼25% loss of water to the subsurface under base flow conditions. The lower measurement point was at an elevation of ∼10 m above sea level and the uppermost measurement point is at ∼30 m. As the surface of the fresh water lens will be very near sea level, the flow path lengths to the aquifer are short, similar to the Honouliuli Stream system and American Samoa analogs. Furthermore, Waimea Stream measurements were conducted on a day when flows were low, suggesting that losses to the aquifer in losing stretches occur more‐or‐less continuously rather than transiently as a result of storm events.

Over the short transport distances between the surface of an aquifer in coastal areas, the likelihood of an interconnected fracture network uninterrupted by an aquitard, usually a fine‐grained ash bed, increases. Indeed, the majority of wells on Oahu in particular and the Hawaiian Islands in general are concentrated near the shoreline (Gingerich & Oki, 2000) where elevations are low and potential travel times and distances to the aquifer are short.

NTM have typical lengths of 1.5–10 μm, and a width of <0.5 μm. Being rod shaped, cells will be aligned with flow directions, so the most critical dimension to bypass auto‐filtration in the aquifer will be the width of the cell ± the width of any attached mineral grain. This suggests that a minimum, continuous fracture aperture network of ∼1 μm between the stream bed and the aquifer must exist for NTM transport. We have assessed the influence of fracture aperture in groundwater travel times using Bear's (Bear, 1972; Konzuk & Kueper, 2004) law for fracture flow. A discussion of the applicable mathematics is presented in supporting information (S2).

**Figure 11:**
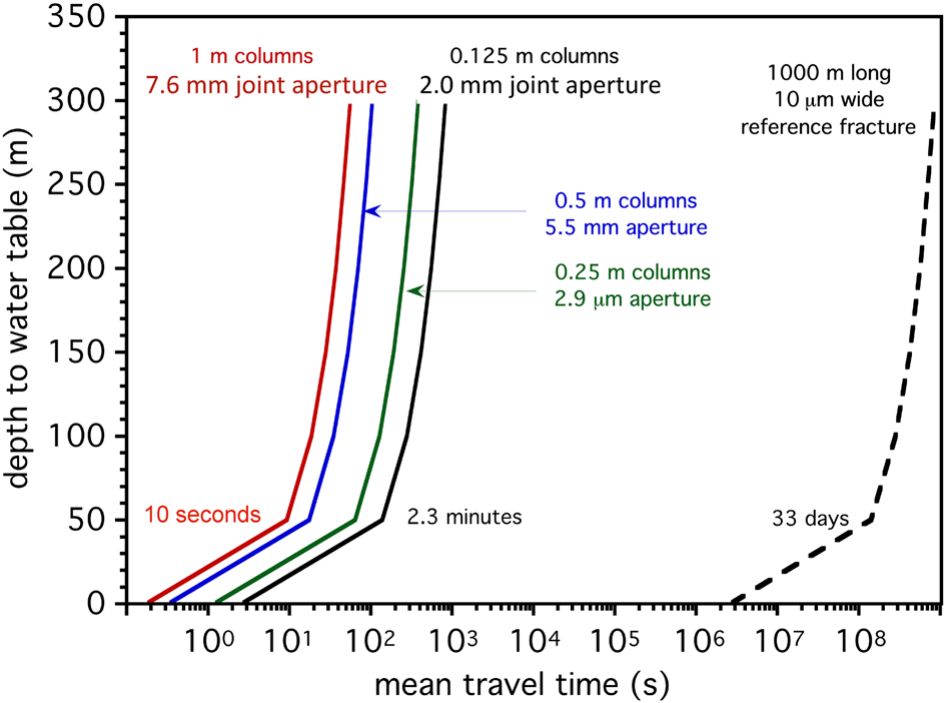
Curves representing the solution to Bear's law (1972) for travel times of water through cooling (columnar) joints of various spacings, similar to the range documented by Goehring (2008), as a function of hydraulic heads (i.e., depth from stream bottom to the aquifer). A relatively narrow reference fracture is presented for comparison. Transit times for colloidal material, including nontuberculous mycobacterial (NTM), can be rapid provided there is an integrated network of fractures between the stream bed and the aquifer. See text for discussion.

It is relatively simple to assess the importance of fracture apertures and travel times in the assessment of NTM transport. We base the calculations on aa flows, typically considered the least permeable rocks in Hawai'i aquifers apart from ash beds (Gingerich & Oki, 2000). Figure 11 presents transport times through the interiors of a'a lava flows, assuming they have idealized hexagonal columns of 0.125, 0.25, 0.5, and 1.0 m widths, a range that is typical of column dimensions measured in the field (Goehring, 2008). Using the composition of average Koolau lava (Nelson et al., 2013), a non‐vesicular lava shrinks by ∼2.6% upon cooling (KWare, 2020; K. Wohletz software author), leading to typical fracture apertures ranging 2.0–7.6 mm over the range of column sizes considered. Larger columns must be separated by wider joints to conserve the volume of shrinkage. A 1 mm fracture aperture is 10^3^ times larger than the 1 μm fracture width required to preclude autofiltration.

The idealized travel times are rapid for all four joint spacing (Figure 11), on the scale of minutes for fracture systems in streams at low elevation (<50 m). The hydraulic conductivity of basalt will not be controlled solely by fractures in aa flows. Rubble zones on the top and bottom of these flows may be even more transmissive (e.g., Doughty, 2000; Pollyea & Fairley, 2012) and ash horizons may be aquitards. Fracture apertures in pahoehoe flows may be much smaller than in a'a due to higher fracture densities upon rapid cooling, but even then they may be perfectly capable of transmitting mineral‐NTM pairs. The (100 m × 10 μm) reference fracture may transmit water on time scales of a month (Figure 11) even if the entire fracture network were this narrow. These calculations are consistent with the empirical observations of Rosa (2017) and Shuler et al. (2019). Furthermore, well‐known size‐exclusion effects for colloids dictate that the calculated travel‐time curves (Figure 11) are minimum values as particles tend to remain in the high‐velocity flow regime between fracture walls, especially at high flow rates (e.g., Auset & Keller, 2004; Keller et al., 2004; McKay et al., 2000).

#### 4.4.2 Transport in the Saturated Zone

The influence of fracture aperture discussed above applies to the saturated zone. Although groundwater flow models should always be approached with appropriate caution, for our model domain, nearly all stream water losses are captured in <95 days with some water reaching production wells in just over a month (Figure 9). How well these calculations represent the true capture and travel times of stream losses is less important than the qualitative observation that stream losses may be readily pulled into active wells. In fact, these time scales are long compared to the appearance of elevated turbidity and coliform bacteria in the wells of American Samoa (Shuler et al., 2019). Thus, the groundwater flow model may provide conservative estimates of travel times.

We know of no specific study that examined the survival of NTM in basalt aquifers. However, Gomez‐Smith et al. (2015) noted that NTM is a major component of thick biofilms in the concrete and iron pipes of municipal water distribution systems. Although pipes are not an aquifer, their study indicated that NTM survives and even thrives in dark, continuously wet, and low‐nutrient environments.

At this time, the mechanisms and rates of NTM transport are necessarily speculative, although we can expect two endmember behaviors. Since NTM prefer to attach to surfaces, they may largely exist as biofilms on fracture surfaces. However, cells may detach and reattach, especially during division. Thus, one transport behavior may mimic a reactive tracer where cell transport rates are slower than the seepage velocity. A second behavior may be exhibited by solitary cells or cells attached to mineral particles, in which case they will be transported at a rate greater than the seepage velocity. Unraveling the behavior of NTM in this regard will be a fruitful avenue of further research.

## 5 Conclusions

In summary, mycobacteria are a significant, if minor component of Hawai'i aquifer and a major component of soil biomes (Kirs et al., 2020), and cell count and transport studies indicate up to >10^7^ cells/ml of bacteria may be expected in fractured basalt. NTM are now known to exist in the riparian environments of Kauai and Oahu where cells may tend to bind with hematite (Glickman et al., 2020). Furthermore, it appears that there are plausible, and possibly likely, pathways for basal aquifers to be continuously inoculated by NTM derived from losing streams and episodically by storm events. Bacteria are capable of transport to water‐supply wells (Figure 1). Being chlorine resistant, NTM may then outcompete other microbes, explaining their prevalence in water distribution systems and home plumbing (Gomez‐Smith et al., 2015; Honda et al., 2016). This would permit NTM, which may be only a minor component of a basalt aquifer biome, to dominate water distribution systems and home plumbing in Hawai'i. Future research of NTM in the natural and built environments should focus on predicting which hydrological and geochemical conditions are most conducive to the survival and transport of these bacteria.

## Conflict of Interest

The authors declare no conflicts of interest relevant to this study.

## Supporting information

Supporting Information S1

Table S1

## Data Availability

Identified NTM species are reported in Table 1, and corresponding sample locations are found in Table S1. Groundwater modeling files (Aquaveo/Modflow format), raw X‐ray diffraction files, and scanning‐electron microscope files can be accessed at: Mendeley Data, V1, https://doi.org/10.17632/mx6vr8bpgn.1.
