# Supplementary material for: Exposure Pathways of Nontuberculous Mycobacteria Through Soil, Streams, and Groundwater, Hawai'i, USA
Source: Geohealth. 2021 Apr 1;5(4):e2020GH000350. doi: 10.1029/2020GH000350 (PMC8025848; doi:10.1029/2020GH000350)
Supplement: Supplementary file 1 — Supporting Information S1 [file GH2-5-e2020GH000350-s002.pdf]

AGU PUBLICATIONS

GeoHealth

Supporting Information for

Exposure Pathways of Nontuberculous Mycobacteria Through Soil, Streams, and Groundwater, Hawai'i, USA

[Stephen T. Nelson[*,1], Schuyler Robinson[1], Kevin Rey[1], Leeza Brown[1], Norm Jones[2], Stephanie N. Dawrs[3], Ravleen Virdi[3], Grant J. Norton[3], L. Elaine Epperson[3], Nabeeh A. Hasan[3], Edward D. Chan[4,5,6], Michael Strong[3], Jennifer R. Honda[3]

[1]Department of Geological Sciences, Brigham Young University
[2]Department of Civil and Environmental Engineering, Brigham Young University
[3]Center for Genes, Environment, and Health, National Jewish Health
[4]Medicine and Academic Affairs, National Jewish Health
[5]Division of Pulmonary Sciences and Critical Care Medicine, University of Colorado Anschutz Medical Campus
[6]Department of Medicine, Rocky Mountain Regional Denver Veterans Affairs Medical Center

Contents of this file

Text S2

Additional Supporting Information (Files uploaded separately)

Caption for Datasets S2: *Formulation of Bear's law as applied in the text*

Introduction

The following text and equations are presented and applied to the discussing of fracture flow from losing streams to the aquifer as discussed in the text. References below are cited in the main article.

* *Corresponding author ;Dept. of Geological Sciences, S-389 ESC, Brigham Young University, Provo, UT 84602; oxygen.isotope@gmail.com; 801-722-5387*

Analytical solutions for fracture flow are relevant for constraining fracture apertures, infiltration depths, and response times. Such a solution is presented for the curves in Figure 11 of the text, where Bear's (1972; Konzuk and Kueper, 2004) law (eq. 2) for fracture flow can be reduced to (eq. 3):

$$Q = -\frac{W\rho e^3}{12\mu}\frac{\Delta h}{\Delta L} = -vA = -vWe \ (2)$$

$$v = \frac{\rho e^2}{12\mu}\frac{\Delta h}{\Delta L} \ (3)$$

$v = \frac{\rho e^2}{12\mu}$ where the fracture length is equal to the hydraulic head (4)

$$Q = flux\ \frac{m^3}{s}$$
$$v = mean\ velocity\ \frac{m}{s}$$
$$W = fracture\ width\ m$$
$$\rho = density\ \frac{kg}{m^3} = 1000\frac{kg}{m^3}$$
$$e = fracture\ aperture\ m$$
$$\Delta h = hydraulic\ head\ m$$
$$\mu = dynamic\ viscosity\ Pa \cdot s = 8.9x10^{-4} Pa \cdot s$$
$$\Delta L = vertical\ fracture\ length\ m$$
$$v = mean\ velocity\ \frac{m}{s}$$
$$A = fracture\ cross - sectional\ area\ m^2$$

A graphical representation for hypothetical travel times to the aquifer for various fracture apertures are shown in Figure 11, where the hydraulic head ($\Delta h = \Delta L$) is assumed to be the vertical distance between the stream surface and the aquifer. As the freshwater lens beneath volcanic islands rises only very gradually inland, and stream elevations are low, travel times may be very short for coastal regions like the Waimea as well as the Honouliuli Stream and American Samoa analogs discussed in the text.
