# Supplementary material for: Exposure Pathways of Nontuberculous Mycobacteria Through Soil, Streams, and Groundwater, Hawai'i, USA
Source: Geohealth. 2021 Apr 1;5(4):e2020GH000350. doi: 10.1029/2020GH000350 (PMC8025848; doi:10.1029/2020GH000350)
Supplement: Supplementary file 2 — Table S1 [file GH2-5-e2020GH000350-s001.pdf]

GeoHealth

Supporting Information for

Exposure Pathways of Nontuberculous Mycobacteria Through Soil, Streams, and Groundwater, Hawai'i, USA

[Stephen T. Nelson*[,1], Schuyler Robinson[1], Kevin Rey[1], Leeza Brown[1], Norm Jones[2], Stephanie N. Dawrs[3], Ravleen Virdi[3], Grant J. Norton[3], L. Elaine Epperson[3], Nabeeh A. Hasan[3], Edward D. Chan[4,5,6], Michael Strong[3], Jennifer R. Honda[3]

[1]Department of Geological Sciences, Brigham Young University
[2]Department of Civil and Environmental Engineering, Brigham Young University
[3]Center for Genes, Environment, and Health, National Jewish Health
[4]Medicine and Academic Affairs, National Jewish Health
[5]Division of Pulmonary Sciences and Critical Care Medicine, University of Colorado Anschutz Medical Campus
[6]Department of Medicine, Rocky Mountain Regional Denver Veterans Affairs Medical Center

Contents of this file

Table S1

Additional Supporting Information (Files uploaded separately)

Captions for Datasets S1: *Details for the stream (and 1 reservoir) sampling events on Oahu and Kauai, Hawaii, summer 2018. UTM Zone 4, NAD83.*

Introduction

The following Table gives sample locations, dates, and times for stream samples discussed in this article. They are included as Supporting Information to: a) decrease the manuscript length, and b) enable the interested reader to locate or revisit these sites as desired.

* *Corresponding author ;Dept. of Geological Sciences, S-389 ESC, Brigham Young University, Provo, UT 84602; oxygen.isotope@gmail.com; 801-722-5387*

Table S1B. Details for the stream (and 1 reservoir) sampling events on Oahu and Kauai, Hawaii, summer 2018. UTM Zone 4, NAD83.

Sample name	Locality/sample type	Sample date	Easting	Northing
Oahu January 2018 samples				
18-STR-1	Manoa Falls Stream	1/22/18 11:44	624357	2359606
18-STR-2	Nuuanu Stream	1/22/18 13:50	622282	2360954
18-STR-3	Kalihi Stream	1/22/18 15:09	618917	2362389
18-STR-6	Waimea Stream	1/23/18 11:47	597716	2392801
18-STR-7	Kahana Stream	1/23/18 15:16	615738	2382418
18-STR-9	Palolo Stream	1/24/18 13:56	625672	2356598
18-STR-10	Waiawa Stream/ditch	1/25/18 9:54	604612	2370911
18-STR-11	Waimano Stream	1/25/18 12:00	609421	2369359
18-STR-12	Wahiawa Reservoir	1/25/18 13:18	598410	2377329
18-STR-13	Maunawili Stream	1/26/18 10:03	628078	2362678
18-STR-14	Maunawili tributary stream	1/26/18 11:13	627800	2362753
18-STR-15	Heeia Stream	1/26/18 14:04	622133	2368077
18-STR-16	Waikane Stream	1/26/18 15:55	619040	2377046
18-STR-17	Kaunala Loop Trail Stream	1/27/18 13:25	602436	2393916
Oahu February 2018 samples				
18-KAR-1	Manoa Falls Stream	2/20/18 16:19	624359	2359611
18-KAR-2	Nuuanu Stream	2/20/18 17:22	622283	2360952
18-KAR-3	Kalihi Stream	2/21/18 14:38	618905	2362376
18-KAR-6	Waimea Stream	2/20/18 10:35	597600	2392897
18-KAR-7	Kahana Stream	2/19/18 10:14	615697	2382402
18-KAR-9	Palolo Stream	2/20/18 15:22	625685	2356576
18-KAR-11	Waimano Stream	2/20/18 13:20	609416	2369363
18-KAR-12	Wahiawa Reservoir	2/20/18 11:41	598416	2377271
18-KAR-13	Maunawili Stream	2/19/18 13:43	628076	2362682
18-KAR-14	Maunawili tributary stream	2/20/18 14:09	627802	2362769
18-KAR-15	Heeia Stream	2/19/18 12:46	622112	2368076
18-KAR-16	Waikane Stream	2/19/18 11:35	619046	2377042
18-KAR-17	Kaunala Loop Trail stream	2/20/18 8:45	602443	2393928
18-KAR-18	Herring Springs	2/21/18 15:40	622191	2358769
Oahu August 2018 samples				
18-KRM-01	Manoa Falls Stream	8/2/18 18:55	624343	2359618
18-KRM-02	Nuuanu Stream	8/3/18 12:42	622296	2360950
18-KRM-03	Kalihi Stream	8/3/18 14:46	618898	2362391
18-KRM-06	Waimea Stream	8/4/18 10:19	597605	2392906
18-KRM-07	Kahana Stream	8/7/18 12:03	615700	2382401
18-KRM-09	Palolo Stream	8/2/18 14:52	625681	2356572
18-KRM-11	Waimano Stream	8/2/18 11:23	609416	2369365
18-KRM-12	Wahiawa Reservoir	8/6/18 10:16	598415	2377330
18-KRM-13	Maunawili Stream	8/3/18 10:31	628068	2362661
18-KRM-14	Maunawili tributary stream	8/3/18 11:20	627793	2362765
18-KRM-15	Heeia Stream	8/3/18 15:57	622145	2368074
18-KRM-16	Waikane Stream	8/7/18 13:51	619040	2377030

18-KRM-17	Kaunala Loop Trail stream	8/6/18 16:25	602449	2393932
18-KRM-18	Herring Springs	8/2/18 16:23	622192	2358768
18-KRM-19	Kamananui Stream	8/4/18 13:43	598686	2392296
18-KRM-20	Kaiwikoele Stream	8/4/18 15:12	598602	2392258
Oahu August 2019 samples				
19-KAR-1	Manoa Falls Stream	July 26-31, 2019	624359	2359611
19-KAR-2	Nuuanu Stream	July 26-31, 2019	622283	2360952
19-KAR-3	Kalihi Stream	July 26-31, 2019	618905	2362376
19-KAR-6	Waimea Stream	July 26-31, 2019	597605	2392906
19-KAR-7	Kahana Stream	July 26-31, 2019	615697	2382402
19-KAR-11	Waimano Stream	July 26-31, 2019	609416	2369365
19-KAR-12	Wahiawa Reservoir	July 26-31, 2019	598415	2377330
19-KAR-13	Maunawili Stream	July 26-31, 2019	628068	2362661
19-KAR-15	Heeia Stream	July 26-31, 2019	622145	2368074
19-KAR-16	Waikane Stream	July 26-31, 2019	619040	2377030
19-KAR-17	Kaunala Loop Trail stream	July 26-31, 2019	602449	2393932
19-KAR-18	Herring Springs	July 26-31, 2019	622192	2358768
Kauai July-August 2018 samples				
18-Kau-19	Waiakoali Stream	7/31/18 16:56	435837	2446770
18-Kau-20	Kawaikoi Stream	7/31/18 17:10	435893	2447521
18-Kau-25	Mohihi Stream	8/2/18 14:47	437986	2445886
18-Kau-26	Unnamed stream 1	8/2/18 16:51	435166	2447559
18-Kau-27	Kauaikinana Stream	8/2/18 17:33	434908	2447662
18-Kau-34	Waimea River	8/3/18 14:49	432114	2430721
Kauai July 2019 samples				
19-Kau-1	Waimea River upstream	7/21/19	433797	2439987
19-Kau-2	Unnamed stream 2	7/21/19	433616	2439999
19-Kau-3	Mohihi Stream	7/22/19	437980	2445881
19-Kau-4	Waiakoali Stream	7/22/19	435827	2446775
19-Kau-5	Kawaikoi Stream	7/22/19	435899	2447499
19-Kau-6	Unnamed stream 1	7/22/19	435145	2447553
19-Kau-7	Kauaikinana Stream	7/22/19	434931	2447659
19-Kau-8	Waimea River	7/22/19	432121	2430699
19-Kau-9	Hanalei River	7/22/19	450989	2456044
